# La imagenología en el diagnóstico y la planificación de sistemas de conductos radiculares: una revisión actualizada

**DOI:** 10.21142/2523-2754-0901-2021-045

**Published:** 2021-03-11

**Authors:** Crismely Taveras Parra, Gustavo Adolfo Fiori-Chíncaro, Ana María Agudelo-Botero

**Affiliations:** 1 Universidad Iberoamericana (UNIBE). Santo Domingo, República Dominicana. dracrismelytaveras@gmail.com Universidad Iberoamericana (UNIBE) Santo Domingo República Dominicana dracrismelytaveras@gmail.com; 2 División de Radiología Bucal y Maxilofacial, Universidad Científica del Sur. Lima, Perú. gfiori@ilaeperu.com Universidad Científica del Sur División de Radiología Bucal y Maxilofacial Universidad Científica del Sur Lima Peru gfiori@ilaeperu.com; 3 Universidad Autónoma de Manizales. Manizales, Colombia. ortoamariabotero@gmail.com Universidad Autónoma de Manizales Universidad Autónoma de Manizales Manizales Colombia ortoamariabotero@gmail.com

**Keywords:** endodoncia, tomografía computarizada de haz cónico, radiología, endodontics, cone-beam computed tomography, radiology

## Abstract

Los sistemas de conductos radiculares presentan una variada conformación morfológica para cada grupo de piezas dentarias. Diversos autores han presentado en diferentes clasificaciones su conformación anatómica y sus variantes. Tenemos los conductos principales, colaterales, laterales, secundario, accesorio, interconducto, recurrente, delta apical y cavo interradicular. La radiología actual aún no permite la visualización de todas estas estructuras; sin embargo, el uso adecuado de las técnicas imagenológicas, junto con el empleo de nuevos equipos 3D por ajustes de parámetros de adquisición con adecuados *software* y algoritmos, permite una precisión mayor en las imágenes lo que favorece la observación de finos detalles útiles para el diagnóstico y abordaje de los tratamientos endodónticos. El objetivo de este artículo es realizar una revisión de la literatura para identificar nuevos conceptos y herramientas imagenológicas útiles para obtener mejores diagnósticos.

## Abstract (en)

Root canal systems present a varied morphological conformation for each group of teeth. Several authors have described the anatomical conformation and its variants in different classifications, including the main, collateral, lateral, secondary, accessory, interduct, recurrent, apical delta and interradicular cavus canals. Currently, radiology still does not allow visualization of all these structures. However, proper use of imaging techniques together with the use of new 3D equipment for adjustments of acquisition parameters with adequate software and algorithms allows greater image precision, facilitating diagnosis and the planning of the most adequate approach for endodontic treatments. The objective of this article was to conduct a review of the literature to identify new concepts and useful imaging tools for better diagnoses.

## INTRODUCCIÓN

Los nuevos estudios demuestran que, por causa de la prevalencia de la distorsión geométrica, la radiografía convencional ofrece un valor limitado en los tratamientos de conductos complejos, lo que lleva al clínico a la obtención de imágenes proporcionadas por una resolución espacial en todos los planos, hoy en día disponible en la tomografía computarizada de haz cónico (TCHC), que proporciona imágenes en 3D que disminuyen el campo de visión (FOV) y aumentan la precisión de la imagen radiográfica ^1^^-^^11^.

La radiología oral está en constante evolución, lo que obliga al clínico a una actualización de sus conocimientos básicos en imagenología. El uso de las TCHC para el dominio y reconocimiento del sistema de conductos radiculares resulta necesario en aquellos casos de abordaje complejo como lesiones postrauma, fracturas radiculares, reabsorciones, fracturas de postes, instrumentos fracturados, calcificaciones radiculares, así como para la planificación y ejecución de cirugías paraendodónticas ^1^^,^^5^^,^^7^^,^^10^^-^^15^.

Sin embargo, existen limitaciones del uso de la TCHC como la presencia de restauraciones dentales, instrumentos fracturados e implantes que provocan ciertas distorsiones que distorsionan el resultado, lo que lleva al clínico a tomar decisiones acerca del equipo y la técnica imagenológica ideal para cumplir con los protocolos y objetivos en un tratamiento de conductos ^5^^,^^10^^,^^11^^,^^14^^,^^15^.

Según la Asociación Estadounidense de Endodoncistas (AAE), la Academia Estadounidense de Radiología Oral y Maxilofacial (AAOMR) y la Sociedad Europea de Endodoncia (ESE), la TCHC solo se recomienda en aquellos casos en los que la radiografía bidimensional no proporciona información suficiente y confiable ^7^^,^^10^^,^^11^^,^^16^^,^^17^. Su valor se reconoce a la hora de realizar los procedimientos cumpliendo estrictamente los principios de ALARA (*as low as reasonably achievable*) ^4^^,^^10^^,^^12^^,^^18^. El objetivo de este artículo es realizar una revisión de la literatura para identificar los nuevos conceptos y herramientas imagenológicas útiles para realizar diagnósticos eficientes.

## MATERIALES Y MÉTODOS 

Se realizó una búsqueda de la literatura en las principales fuentes de información: Medline (vía PubMed), Elsevier, SciELO y LILACS, y se usaron los términos de búsqueda con una limitación de fecha de los últimos 10 años. Los artículos seleccionados debían incluir información referente a endodoncia, tomografía computarizada de haz cónico y radiología

## MORFOLOGÍA DE LOS SISTEMAS DE CONDUCTOS RADICULARES

La terapia endodóntica se basa en el manejo de la morfología de los conductos radiculares y sus posibles cambios relacionados con caries, trauma, enfermedad periodontal y aquellos propios de la edad ^10^^,^^19^^-^^24^.

La variación anatómica y la simetría bilateral de los conductos pudieran estar asociados con factores étnicos, la edad y el sexo del paciente. Dentro del sistema de conductos radiculares consideramos la presencia de conductos principales, colaterales, laterales, secundarios, accesorios, interconducto, recurrente, delta apical y cavo interradicular. A lo largo de los años, mediante distintos métodos de radiografía convencional, se ha presentado una variedad en morfología de conductos por cada grupo de diente, clasificados por reconocidos autores que han estudiado a fondo su dinámica. Wein (1999) clasificó los conductos radiculares en cuatro variedades, Vertucci (1984) determinó 8 tipos y, posteriormente, Sert y Byirli (2004) rectificaron las propuestas por Vertucci, y establecieron la clasificación la más aceptada hoy en día ^19^^,^^20^^,^^22^^,^^25^^,^^26^. 

Varias radiografías intraorales en distintas angulaciones no garantizan la identificación de toda la anatomía. La TCHC permite observar los sistemas de conductos desde varios ángulos axiales y sagitales, a pesar de la superposición de estructuras anatómicas que pudiesen interferir y ocultar información en la determinación del diagnóstico endodóntico. No obstante, pese a las complejidades presentadas en cada caso en particular, el conocimiento y la experiencia del clínico juega un papel primordial en la determinación de la entidad endodóntica presentada ^20^^,^^21^^,^^27^.

## TÉCNICAS RADIOLÓGICAS USADAS ACTUALMENTE EN EL DIAGNÓSTICO Y LA PLANIFICACIÓN DE TRATAMIENTO DE CONDUCTOS RADICULARES

El empleo de distintas técnicas radiográficas tiene el objetivo de determinar los siguientes casos: identificar la forma de las raíces y conductos superpuestos; ubicación, tamaño y dirección de curvaturas apicales; relación con estructuras anatómicas; patologías apicales; iatrogenias (instrumentos fracturados, perforaciones); traumas y reabsorciones dentales ^28^^-^^30^.

Una técnica continuamente empleada en endodoncia es la angulación vertical de la bisectriz; no obstante, presenta riesgos de distorsión como método preoperatorio de diagnóstico, por lo que ha sido modificada para lograr el paralelismo, en conjunto con el uso de radiografías en distintas angulaciones que favorecen los resultados y disminuyen la superposición anatómica ^28^^,^^29^.

La técnica de exploración triangular propuesta por Bramante en 1980 es de gran utilidad para observar errores iatrogénicos como escalones, perforaciones y desvíos, mediante la toma de tres radiografías dentales: una de forma estándar, otra con angulación mesial y otra con angulación distal. Tiene como fundamento que la visualización de los defectos es imposible cuando se superponen sobre el espacio del conducto ^28^.

La confirmación del proceso osteolítico durante el diagnóstico requiere una correcta elección de la técnica radiográfica. La revisión de la literatura coincide en que las radiografías convencionales carecen de sensibilidad para determinar patologías apicales, por el alto ruido que provoca la presencia de estructuras anatómicas, lo cual demuestra que, entre las imágenes 2D, las más certeras son aquellas de tipo digital. En la revisión actual, el 90% de la literatura ofrece datos favorables para el uso de TCHC de campo pequeño; sin embargo, las lesiones apicales de gran tamaño (entre 1.4 mm-1.8mm de diámetro) no presentan diferencias significativas respecto de la técnica imagenológica empleada para su visualización ^31^^-^^36^.

## USO DE LA TCHC EN EL DIAGNÓSTICO ENDODÓNTICO

En 1990, para producir exploraciones tridimensionales del esqueleto maxilofacial con una dosis de radiación considerablemente menor que la tomografía compu-tarizada convencional (CT), surgió un sistema tipo escáner extraoral conocido con el nombre de tomografía computarizada de haz cónico, de ampliación geométrica, que disminuye la magnificación, distorsión, superposición y tergiversación de las estructuras dentales ^4^^,^^29^^,^^31^^,^^37^.

Tachibana y Matsumoto, también en 1990, fueron los primeros en destacar sus virtudes en el área de la endodoncia, con el fin de superar los ruidos anatómicos y la distorsión geométrica al momento de diagnosticar las enfermedades pulpares, revelando información de la morfología del conducto, estructuras anatómicas próximas, grosor de corticales y presencia de la lesión periapical. El campo de vision (FOV) mide el volumen de exploración de una unidad TCHC en particular, conformado por el tamaño, la forma del detector, la geometría de la proyección del haz y la colimación, lo que limita la exposición a la radiación de una región particular de interés ^12^^,^^35^^,^^36^.

Existe mucha controversia con relación al número de proyecciones y su relación en la calidad de la imagen. Los tomógrafos con un volumen pequeño disponen de dosis más bajas, similares a las de 2 a 7 radiografías periapicales, mientras que la dosis de radiación en equipos de gran volumen es similar a la de una serie de periapicales completa. El protocolo de la TCHC es determinado por parámetros de precisión tales como especificidad, sensibilidad, predicción, voltaje, corriente del tubo y filtros posteriores al tiempo adquisición, afectados por objetos metálicos que se superponen a la raíz del diente y pudiese imitar fracturas radiculares ^38^^-^^42^.

Estudios han demostrado resultados positivos haciendo uso de campos de visión (FOV) pequeños como 3 x 4, 3,7 x 5, 4 x 4, 4 x 8, 5 x 5, 5 x 8, 6 x 6, 7,5 x 8,1, 7 x 8, 8 x 7,5, 8 x 8, 8,5 x 8,5 (con vóxel de 0,2 µm) y 16 x 6 (con vóxel, 0,25 µm), con valores de sensibilidad y especificidad ligeramente mejores en comparación con otros protocolos, que van desde un vóxel de mayor tamaño: de 0,60 µm a 0,86 µm, para un campo de visión 8 x 8, y de 0,80 µm a 0,53 µm, para un campo de visión 16 x 6. Esto mejora la observación de detalles finos en piezas dentales (asociado a las estructuras de soporte) y reduce la presencia de artefactos metálicos, lo que favorece una observación confiable y mejora los resultados ^41^^,^^43^ (Anexo 1).

El vóxel describe la parte más pequeña de una imagen. Al ser isotrópico, permite la reconstrucción en cualquier plano con resoluciones variables que oscilan entre 0,40 mm y 0,07 mm, con un promedio de 0,15 mm, el cual es levemente más bajo que el tamaño de un píxel de un tomógrafo convencional. Autores como Bechara refieren que el número de fotones adquiridos por un vóxel daría como resultado una disminución en la señal emitida, lo que conduce a un aumento en el ruido y provoca una resolución espacial más baja en un vóxel de tamaño grande, no recomendable para observar detalles finos como la detección de lesiones óseas en periodontitis apicales. Mientras más reducido sea el campo visual mayor será la resolución; los equipos de última generación permiten que este signo sea visible ya que la resolución nominal de sus vóxeles varía de 0,40 mm a 0,07 mm ^40^^,^^41^^,^^45^.

Con la tridimensionalidad dental es posible el reconocimiento de la distancia entre la constricción apical y el ápice anatómico, gracias a técnicas de tomografía microcomputarizada (Micro-CT), que proporcionan información sobre estructuras menores, lo cual favorece una instrumentación idónea, una adecuada remoción microbiana y la eliminación del ruido por ajustes de parámetros de adquisición que absorben la radiación baja de energía y reducen el efecto del endurecimiento del haz. En un estudio reportado por Burch y Hullen, el 92,3% de los casos la distancia promedio desde el ápice radiográfico hasta el foramen apical es de 0,59 mm. Dummer propuso que la distancia entre la unión cemento-dentinal y el foramen apical es de 0,51 mm en jóvenes y 0,79 mm en adultos mayores ^46^^-^^49^.

Un nuevo programa de *software* de análisis mediante imágenes de TCHC, llamado E-Vol DX (CDT Software, Bauru, SP, Brasil), determina la posición del foramen con la configuración de un conjunto de filtros almacenados en grupos, lo cual mejora la estandarización de brillo y contraste en las imágenes, incluida la opción de capturar una resolución de 192 dpi con una opción de 384 dpi, equivalentes a la cantidad en número de píxeles que hay en una pulgada de una imagen reproducida en la pantalla de un ordenador ^5^^,^^47^^,^^49^.

El *software* E-Vol DX facilita el análisis de imágenes CBCT a través de estrategias dinámicas de lectura en formato de imagen digital y comunicaciones en medicina (lectura DICOM), usando cortes axiales secuenciales de cada raíz y navegación de la imagen de coronal a apical usando cortes axiales de 0,1mm / 0,1mm. Este movimiento longitudinal ofrece la posición exacta del foramen apical en relación con otras estructuras en cortes CBCT axiales, sagitales y coronales de 0,1 mm/0,1 mm desde el tercio cervical radicular hasta el ápice radicular ^5^^,^^47^.

La utilidad de la TCHC en la identificación de conductos está asociada con las bajas dosis de radiación efectiva sin distorsiones que algunos equipos proporcionan, con tamaño de vóxel de 76 µm útiles en la localización de canales, ya que histológicamente el grosor de un canal promedia los 100 µm; aunque difiere de otros donde basado en el Teorema de Muestreo de Nyquiest es preciso tamaños de vóxel de 50µm o menos. Según algunos estudios, bajo este sistema de medición, el diámetro de los canales accesorios muestra un rango de 10-180 µm, con un diámetro prevalente de 20-30 µm. Un estudio realizado por Paras mostró un diámetro de 6 a 43µm y de 4 a 300 µm en las áreas de furca ^5^^,^^40^^,^^45^^,^^47^^,^^50^^,^^51^.

## CONCLUSIONES

La tomografía computarizada de haz cónico ha promovido cambios en los enfoques de endodoncia y mejorado la toma de decisiones en casos clínicos complejos, basados en la correlación clínica e imagenológica. La tecnología tiene mucho que aportar, pero también tiene un límite de dificultades respecto de lo que quisiéramos observar. La interpretación de la imagen adquirida puede verse comprometida por el método de visualización del *software* elegido, que muchas veces tiene herramientas de navegación limitadas y carece de filtros adecuados para superar ciertos desafíos. 

En la evaluación de estructuras dentarias para casos de análisis de conductos radiculares y sus variantes, casos de fracturas, fisuras, reabsorciones, etc., aún no pueden ser visualizados detalles definidos sobre todo en estructuras o dimensiones por debajo de 100 µm; sin embargo, el uso adecuado de las técnicas imagenológicas, junto con el empleo de nuevos equipos 3D por ajustes de parámetros de adquisición con adecuados *software* y algoritmos, permite mejorar el resultado final de las imágenes.

Al ser los tratamientos de conductos un tratamiento electivo, es importante que el odontólogo haga parte de su práctica el manejo de las diferentes técnicas imagenológicas y conozca las herramientas con que se cuenta actualmente y el futuro aporte de la tecnología en su desarrollo clínico y profesional.

## Fuente de financiamiento:

Autofinanciado

## ANEXO 1

**Tabla 1:** Campo de visión y vóxel ideal para la visualización de conductos radiculares

Conducto radicular	Campo de visión (FOV)	Vóxel
Conducto principal	3 x 5	0,25 mm
	3 x 6	0,25 mm
	4 x 4	0,08 mm
	4 x 8	0,25 mm
	5 x 5	0,11 mm
	6 x 6	0,25 mm
	7 x 8	0,25 mm
	8 x 7	0,25 mm
	8 x 8	0,25 mm
Conducto accesorio	3 x 5	0,25 mm
	3 x 6	0,25 mm
	4 x 4	0,08 mm
	4 x 8	0,25 mm
	5 x 5	0,11 mm
Conducto lateral	3 x 5	0,25 mm
	3 x 6	0,25 mm
	4 x 4	0,08 mm
	4 x 8	0,25 mm
	5 x 5	0,11 mm
Conducto interradicular	3 x 5	0,25 mm
	3 x 6	0,25 mm
	4 x 4	0,08 mm
Conducto colateral	3 x 5	0,25 mm
	3 x 6	0,25 mm
	4 x 4	0,08 mm
	4 x 8	0,25 mm
	5 x 5	0,11 mm
Deltas apicales	3 x 6	0,25 mm
	3 x 5	0,25 mm
	4 x 4	0,08 mm
